# High-Resolution Microbial Community Succession of Microbially Induced Concrete Corrosion in Working Sanitary Manholes

**DOI:** 10.1371/journal.pone.0116400

**Published:** 2015-03-06

**Authors:** Alison L. Ling, Charles E. Robertson, J. Kirk Harris, Daniel N. Frank, Cassandra V. Kotter, Mark J. Stevens, Norman R. Pace, Mark T. Hernandez

**Affiliations:** 1 Department of Civil, Environmental, and Architectural Engineering, University of Colorado Boulder, Boulder, CO, 80309, United States of America; 2 Department of Molecular, Cellular, and Developmental Biology, University of Colorado Boulder, Boulder, CO, 80309, United States of America; 3 Department of Pediatrics, University of Colorado School of Medicine, Aurora, CO, 80045, United States of America; 4 Department of Infectious Diseases, University of Colorado School of Medicine, Aurora, CO, 80045, United States of America; Missouri University of Science and Technology, UNITED STATES

## Abstract

Microbially-induced concrete corrosion in headspaces threatens wastewater infrastructure worldwide. Models for predicting corrosion rates in sewer pipe networks rely largely on information from culture-based investigations. In this study, the succession of microbes associated with corroding concrete was characterized over a one-year monitoring campaign using rRNA sequence-based phylogenetic methods. New concrete specimens were exposed in two highly corrosive manholes (high concentrations of hydrogen sulfide and carbon dioxide gas) on the Colorado Front Range for up to a year. Community succession on corroding surfaces was assessed using Illumina MiSeq sequencing of 16S bacterial rRNA amplicons and Sanger sequencing of 16S universal rRNA clones. Microbial communities associated with corrosion fronts presented distinct succession patterns which converged to markedly low α-diversity levels (< 10 taxa) in conjunction with decreasing pH. The microbial community succession pattern observed in this study agreed with culture-based models that implicate acidophilic sulfur-oxidizer *Acidithiobacillus* spp. in advanced communities, with two notable exceptions. Early communities exposed to alkaline surface pH presented relatively high α-diversity, including heterotrophic, nitrogen-fixing, and sulfur-oxidizing genera, and one community exposed to neutral surface pH presented a diverse transition community comprised of less than 20% sulfur-oxidizers.

## Introduction

Microbially induced concrete corrosion (MICC) significantly reduces the operational life of sewage conveyance and treatment works and costs billions of dollars for replacement and remediation annually [1,2]. The occurrence of MICC has prompted numerous studies of associated microbes using conventional culture-based methods [3–6] or molecular community analysis [7–12]. Because of the time frames and access (dangerous confined spaces) involved, most MICC studies have focused on characterizing microbes in areas with advanced corrosion. Less attention has been directed to how microbes in these environments develop into communities associated with chronic corrosion. Due to a gaseous inorganic substrate (hydrogen sulfide) and steep pH gradients (< pH 1), these unique engineered environments present extreme selection pressures which support low diversity communities This low diversity and staged pH depression makes MICC environment a good model for microbial community succession.

Microbially induced corrosion of concrete is caused by the dissolution of cement (calcium carbonate binder) colonized by an acid-producing autotrophic microbial community [13,14]. Based on culture-based observations, corrosion has been modeled as a stepwise process mediated by pH reduction. After surface pH is abiotically lowered by acidic gases, neutrophilic organisms colonize the surface and are succeeded by acidophilic organisms as the pH decreases further [5,6,15,16]. These acidogenic sulfur-oxidizing microbes enriched on concrete surfaces above the water line oxidize hydrogen sulfide gas to elemental sulfur and sulfuric acid. The corrosion caused by these autotrophic communities also appears to be influenced by carbon dioxide abundance [12].

While the succession of microbial communities involved in concrete corrosion has been hypothesized for over 20 years, the literature in this arena is dominated by culture-based studies, which typically account for less than 1% of environmental community members [17]. These studies remain the accepted models in the wastewater industry, yet there is little corroboration from molecular observations in these environments. Studies using phylogenetic rRNA techniques have revealed the occurrence of organisms with diverse metabolic types in corroding concrete [7–11,18]. The prevalence of organism other than sulfur-oxidizers remains poorly characterized, especially at early stages of corrosion where pH selection pressures are less severe. A single report has attempted longitudinal phylogenetic analyses of microbial DNA recovered from corroding concrete, from a single location, across three sampling dates [9]. The majority of current microbial community analyses leverage high-throughput sequencing techniques (e.g. 454 or Illumina pyrosequencing) that produce large numbers of short sequences. However, sequence databases used in microbial ecology to identify the taxonomy of sample sequences are built on long sequences obtained using Sanger capillary sequencing, and future microbial ecology work will continue to require long sequences for databases.

The goal of this study was to investigate changes in the relative abundances of concrete-associated microbes over time. To elucidate community development, communities were monitored over a timespan ranging from initial placement in a corrosive environment through the occurrence of severe corrosion. In the environment studied, this corresponded to one year of longitudinal monitoring. Eighteen concrete specimens were suspended above domestic wastewater flowing through two manholes on the Colorado Front Range. The atmospheres in these manholes were monitored for several years prior to this campaign, and they were shown to be aggressive corrosion environments. At each predetermined sampling date (exposures up to one year), specimens were removed and corroded material was analyzed. Microbial community analysis for each time point was performed using sequencing and phylogenetic analysis of 16S rRNA gene V1–V2 region amplicons on the Illumina MiSeq platform to assess bacterial sequences and universal V4–V7 region SSU rRNA clones on the Sanger platform for a multi-domain perspective and cross-platform comparisons.

## Methods

### Field experiments

A common structural concrete formulation was chosen and mixed according to ASTM standard C192 using Type II Portland cement, pea gravel, and sand. Casted specimens were cured at 90% relative humidity for 24 hours before their molds were removed. There were three consecutive and independent periods during which concrete specimens were installed, recovered at pre-determined intervals (Table 1), and analyzed. For each of these longitudinal experiments, 2.5 cm concrete cubes were exposed in manhole headspaces for between one and ten months. For the longest sampling points (one year), concrete cylinders (7.5 cm diameter x 15 cm) were used because accelerated corrosion put the smaller cubes at risk for physical recovery. All specimens were suspended by plastic cord or stainless steel bolts in one of two manholes with high headspace concentrations of hydrogen sulfide (>300 ppm), which were known to provide accelerated corrosion environments. These manholes were situated approximately 100 meters from each other on a 1.8 meter fiberglass interceptor, designated Manhole 1 (MH1) and Manhole 2 (MH2). 18 samples were collected from all 18 of the exposed specimens (6 each in Experiments 1, 2A, and 2B). Limited manhole volume constrained specimen recovery to one individual cube on each sampling date (irreversibly and destructively sampled). As a result, the data had some variance, as each specimen sampled was an independent observation with no replication. However, the large number of sampling events and the observation of consistent trends across all experiments mitigated this lack of experimental replication.

**Table 1:** Field Experiment Schedule.

		2011			2012											
Experiment	Manhole	O	N	D	J	F	M	A	M	J	J	A	S	O	N	D
1	MH1 (100 to 500 ppm H_2_S)			I		X		X		X		X		X		X
2A	MH2 (200 to 500 ppm H_2_S)											I	XX	X	XX	X
2B	MH2 (200 to 500 ppm H_2_S)	I		X		X		X		X		X		X		

I indicates initial installation date. X’s indicate sampling date.

### Sampling methodology

Hydrogen sulfide, carbon dioxide, methane, and oxygen levels in manhole headspaces were measured using a GasAlert Micro5 IR gas monitor (BW Technologies, Lincolnshire, IL) calibrated every 90 days. Gas measurements were taken prior to opening each manhole through a sampling tube inserted into manhole cover using the monitor’s attached pump module. All gas measurements were conducted between the hours of 11am and 3pm. On sampling dates, specimens were transported to the laboratory in foil-covered beakers rendered sterile and DNA-free by burning at 550°C for 3 hours. At the laboratory, corroded material and surface deposits (corrosion product) was scraped into burned ceramic crucibles using sterile metal spatulas. Samples for DNA analysis were collected in 3 mL of 1M Tris/0.01mM EDTA buffer to minimize DNA damage and to complex metal ions that could potentially inhibit DNA amplification.

### Physio-chemical parameters

The pH of corrosion products was measured by adding a known amount of purified (membrane-filtered, de-ionized) water (pH 6–8) to a known mass of sample. The mixture was allowed to equilibrate for an hour, and then pH was measured. Original pore water pH was calculated based on the amount of sample, the amount of water added, and the original pH of the water. To determine moisture content, corrosion products were dried at 60°C in ceramic crucibles for at least 8 hours, and moisture content was calculated by dividing water mass lost by the original (wet) mass. Following the removal of corroded material, concrete specimens were cleaned, rinsed, and dried for 48 hours at 6000B0030C, then massed. These masses were compared to the original mass of each specimen before manhole exposures.

### DNA extraction

Buffered samples were kept at 4°C for a maximum of 24 hours before DNA extraction in 1M Tris/0.01mM EDTA buffer. Samples were vortexed to dislodge cells from corrosion matrix and 800 mL of buffer was removed for DNA extraction. 500 mL NaCl/TE buffer with SDS, 250 mL phenol, 250 mL chloroform, and 0.1 mm silica/zirconium beads were added and the mixture was bead-beaten. DNA was precipitated from the supernatant using glycogen, ammonium acetate, and isopropanol [19]. The pellets were washed with 70% ethanol and re-suspended in sterile DNase/RNase-free water (Fisher Scientific). Extracted, eluted DNA was stored at -80°C until further analysis. DNA analysis by each method (Illumina MiSeq V1V2 and Sanger clones) was conducted for one DNA aliquot from each sample, with the following exceptions: a.) extracted DNA from one sample in Experiment 1, one sample in Experiment 2A, and two samples in Experiment 2B were not successfully amplified for either platform, and b.) extracted DNA from three additional samples in Experiment 1 and one additional sample in Experiment 2A was insufficient for Sanger analysis.

### 16S rRNA V1V2 region Illumina MiSeq amplicon sequencing and analysis

Bacterial community composition was analyzed using Illumina MiSeq personal sequencing platform to sequence 16S ribosomal RNA amplicons, which were generated following previously described methods using broad-range PCR primers (27F and 338R) that amplify approximately 350 bp of the V1V2 variable region [20]. Primers were barcoded [21] and modified with adapter sequences required for the Illumina MiSeq platform. PCR amplicons from each sample were normalized based on agarose gel densitometry, pooled in approximately equal amounts, and the resulting mixture was gel-purified to select the correctly sized amplicon products as previously described [22], and the gel-purified amplicons were concentrated using a DNA Clean and Concentrator Kit (Zymo, Irvine, CA). Pooled amplicons were quantified using Qubit Fluorometer 2.0 (Invitrogen, Carlsbad, CA), and each pool was diluted to 2 nM and denatured with 0.2 N NaOH at room temperature. The denatured DNA was diluted to 15 pM and spiked with 25% of Illumina PhiX control DNA prior to loading the sequencer. Illumina paired-end sequencing was performed on the MiSeq platform with version 2.0 of the MiSeq Control Software, using a 500-cycle version 2 reagent kit.

Illumina MiSeq paired-end sequences were sorted by sample via barcodes in the paired reads with a python script. The sorted paired reads were assembled using phrap [23,24]. Assembled sequence ends were trimmed over a moving window of 5 nucleotides until average quality met or exceeded 20. Trimmed sequences with more than 1 ambiguity (any non-specific base call) or shorter than 200 bp were removed from analysis. Potential chimeras were identified with Uchime (usearch6.0.203_i86linux32) [25] using the Schloss SILVA reference sequences [26] and removed from subsequent analyses. Assembled sequences were aligned and classified with SINA 1.2.11 [27] using the 418,497 bacterial sequences in Silva 115NR99 [28]. Operational taxonomic units (OTUs) were produced by clustering sequences with identical taxonomic assignments, and these taxa groups were used in further community analyses. Explicet v2.8.4 (www.explicet.org) [29] was used for display, analysis, and figure generation of both Illumina and Sanger sequence data. This process generated 270,576 sequences of average length 300 nt. The average number of sequences per sample was 19,276 (minimum of 9,818, maximum of 29,617).

The median Goods coverage score for libraries, a measure of completeness of sequencing, was ≥ 99%, indicating that the depth of sequencing was sufficient to fully describe the biodiversity of the samples [30,31]. Alpha-diversity (sample richness as estimated by Chao1 [32]) was calculated in Explicet at the rarefaction point of 4,478 sequences with 1,000 bootstrap re-samplings. 95% confidence intervals were calculated based on these 1,000 results.

### 16S rRNA clone Sanger sequencing and analysis

Extracted genomic DNA was amplified for cloning by PCR with (nominally) universal SSU rRNA gene primers 515F and 1391R. PCRs were conducted at 94°C for 2 min, followed by 30 cycles of 94°C for 20 s, 52°C for 20 s, and 65°C for 1.5 min, followed by a 65°C elongation step for 10 min. Each 50 μL reaction mixture contained 10 μL Eppendorf 2.5X HotMasterMix (Eppendorf, New York, NY), 10 μL water, 0.05% bovine serum albumin (Sigma-Aldrich, St. Louis, MO), 100 ng of each oligonucleotide primer, and 1 to 5 ng of template DNA. Triplicate PCRs were conducted for each sample and pooled before purification with the Montage gel purification system (Millipore). PCR-amplified DNA genes were cloned into TOPO TA vectors and transformed into electrocompetent TOPO One-shot cells (Life Technologies, Carlsbad, CA) using a Transporator Plus electroporator (BTX, Holliston, MA). Transformed cells were grown overnight at 37°C on LB-agar with 1% ampicillin sodium-salt (Sigma-Aldrich, St. Louis, MO), and 96 colonies per sample were picked and prepared for sequencing. Cells were boiled to release DNA and the resulting supernatant was amplified using T3/T7 primers targeting the TOPO vector. T3/T7 PCR product was cleaned using ExoSAP-IT (Affymetrix, Santa Clara, CA). Prepared DNA was sequenced from both ends with capillary electrophoresis using an Amersham MegaBACE 1000 Sanger sequencer (GE Healthcare, Waukesha, WI) or using an ABI PRISM 3730xl sequencer (Applied Biosystems, Carlsbad, CA) using BigDye Terminator Sequencing Kit v3.1 via Beckman Coulter Genomics (Danvers, MA).

Amersham MegaBACE chromatograms were converted to base-calls with phred [23,24]; vendor base calls were used for ABI PRISM generated sequences. T3/T7 reads were assembled, screened for vector contamination using CrossMatch with Univec [23,33,34], then trimmed and checked for chimeras as described in Section 0. Trimmed sequences with more than 1 ambiguity or shorter than 500 bp were discarded. Sequences were inserted into the guide tree of Silva 115NR99 [28] and taxonomy calls were generated via NDS export with ARB [35], and operational taxonomic units (OTUs) were produced by clustering sequences with identical taxonomic assignments.

### Statistical testing

Linear regression models were developed using R Statistics Program [36] to relate exposure time to community composition and corrosion severity. Pairwise comparisons of linear regression line slopes were compared using analysis of covariance (ANCOVA). Individual microbial groups that differed in prevalence or abundance between treatment groups were identified through a two-part statistical test that accounts for zero-inflation and non-normal distributions [37].

### Sequence database submission

Raw paired-end Illumina MiSeq reads were submitted to the NCBI Small Read Archive under BioProject PRJNA201330. Assembled Sanger sequences were submitted to GenBank under accession numbers KF844359 through KF845940.

## Results and Discussion

### Tracking corrosion severity

**Fig 1:**
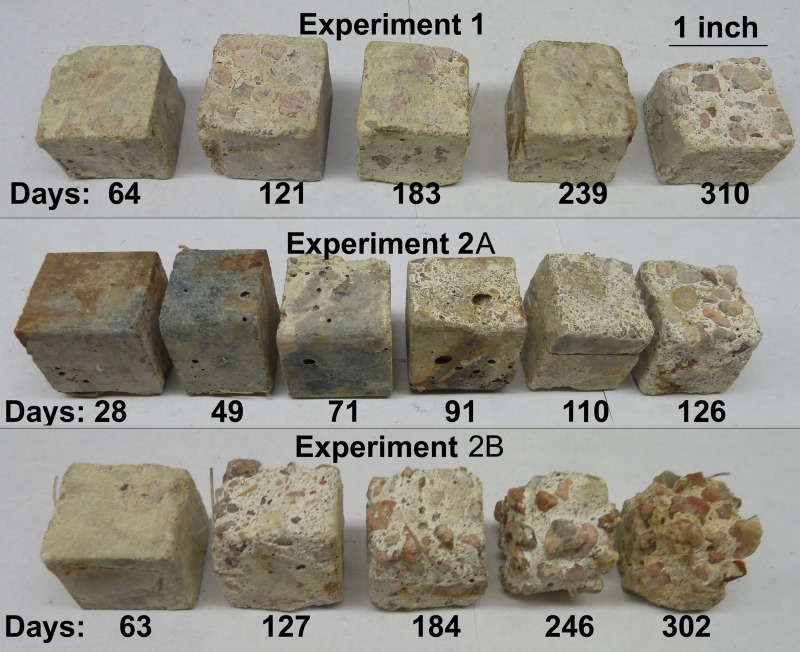
Cubic concrete specimens after exposure to corrosive environments in a wastewater manhole. Corroded material was removed and specimens were dried prior to photographing.

As judged by regression of gravimetric data and surface area assessments specimens exposed to the atmosphere in MH2 (Experiments 2A and 2B) experienced more mass loss and surface corrosion than those exposed to the atmosphere in MH1 (Experiment 1) (ANCOVA p<0.01 for both parameters). In all cases hydrogen sulfide levels were significantly higher in MH2 than the downstream MH1. Lower hydrogen sulfide gas concentrations in MH1 likely facilitated slower diffusion of hydrogen sulfide to the concrete surface moisture, resulting in reduced amounts of substrate for acidogenic bacteria. Fig. 1 shows each specimen after removal of corrosion product.

### Alpha diversity and pH over time

**Fig 2:**
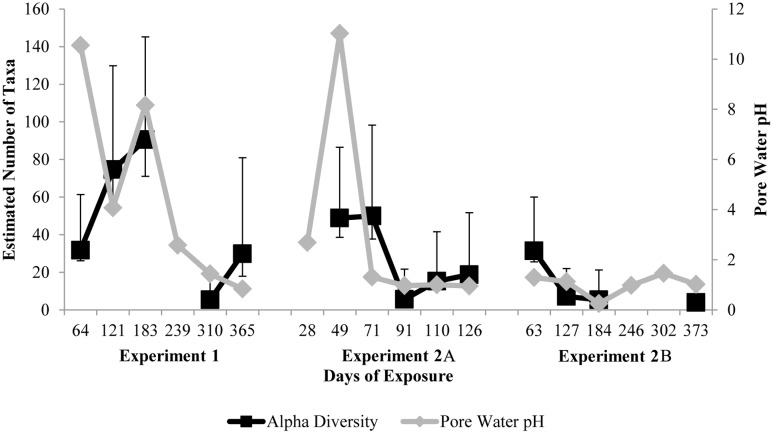
Estimated bacterial community α-diversity in corrosion products as judged by Chao1 using Illumina MiSeq V1V2 16S amplicon sequencing (black square) and estimated pore water pH (grey diamond) after one to twelve months of exposure in a manhole environment. Error bars indicate 95% confidence intervals for 1,000 bootstrap calculations of Chao1.

Bacterial α-diversity on samples in MH2 consistently decreased over the course of each experiment (regression p<0.1) (Fig. 2). This trend coincides with a rapid drop in pore water pH (Fig. 2). In specimens from MH1 (Experiment 1), pore water pH dropped at a slower rate than in MH2, and α-diversity increased for six months before eventually decreasing. In MH1, the transition from alkaline, fresh concrete to acidic, corroded concrete was more gradual. This resulted in sustained neutral pore water pH, which selected for a heterogenous neutrophilic microbial community. The estimated α-diversity dropped below 20 taxa after three months in MH2 or ten months in MH1. This is atypically low for environmental microbial communities [38], and it illustrates the extreme conditions present that are parallel to water quality conditions in acid mine drainage enviroments [39]. Selection pressure from one primary energy source (H_2_S) and very acidic conditions (pH<2) likely caused very low α-diversity in very close proximity to domestic wastewater.

### Microbial succession by Illumina bacterial V1V2 amplicon sequencing

**Fig 3:**
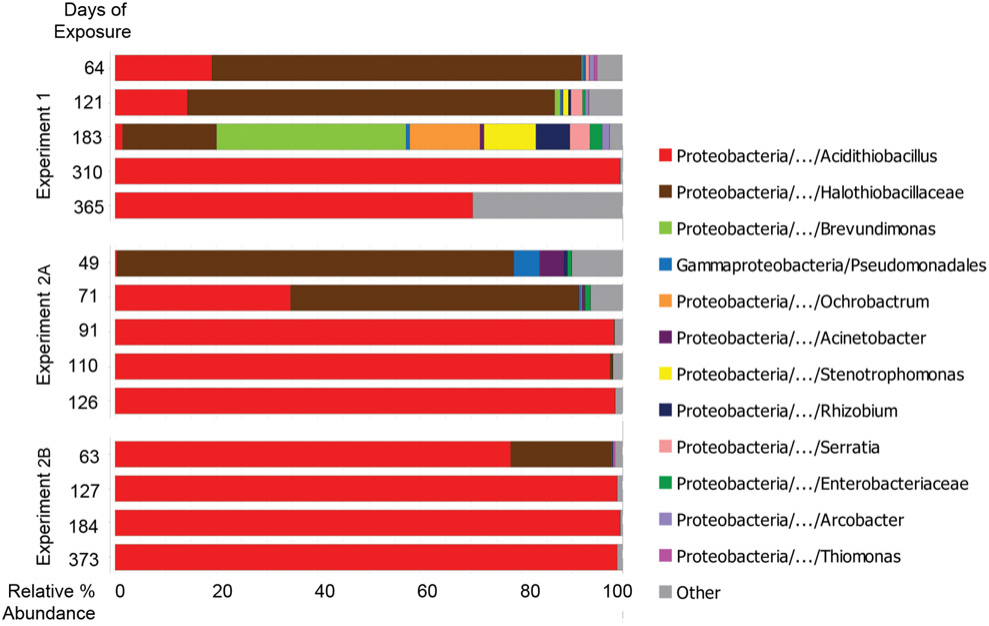
Bacterial taxa observed in concrete surfaces and in corrosion products during three manhole monitoring campaigns by Illumina MiSeq of V1V2 rRNA amplicons. Taxa with greater than 1% representation in any sample are shown. Bar widths indicate relative percent abundance of microbial taxa in sample libraries; bar colors indicate taxa identity.

Bacterial community analysis was conducted using Illumina sequencing of 16S rRNA V1V2 region amplicons. In each experiment, neutrophilic genera were succeeded by acidophilic genera as pore water pH decreased with extended exposure. Early communities (<6 months in MH1 and <2 months in MH2) exhibited high relative abundance of *Halothiobacillus* spp., a neutrophilic sulfur-oxidizing bacteria. As pH values dropped below 2, regardless of location, communities were dominated (>95%) by acidophilic sulfur-oxidizer *Acidithiobacillus* spp. (linear regression p-value < 0.01) (Fig. 3). This trend corresponds to communities previously observed in this environment [7–12].

Early stage communities had higher α-diversities and larger abundances of taxa related to heterotrophic genera, such as *Pseudomonas*, *Serratia*, and *Stenotrophomonas*. Twenty-four genera, mostly related to heterotrophs, were significantly more abundant before 100 days of exposure than after 100 days of exposure (two-part p<0.05). In Experiment 1, near neutral pH levels (8.2) were still observed after 183 days of exposure; specimens removed at that time hosted a diverse spectrum of heterotrophic bacterial types including *Brevundimonas* spp., *Ochrobactrum* spp. *Stenotrophomonas* spp., and *Rhizobium* spp. The genera *Ochrobactrum* and *Stenotrophomonas* include opportunistic human pathogens, and genera *Ochrobactrum* and *Rhizobium* include endosymbiotic nitrogen-fixers [40–42]. This community is unlike any other community reported associating with microbially induced concrete corrosion; it illustrates significant community structure changes that can occur in ephemeral environmental conditions (e.g. neutral pH). The extended conditions of neutral pH likely facilitated the development of a more heterogenous neutrophilic microbial community on the MH1 specimens. In the following months, reflected by subsequent samples in the MH1 time series (Experiment 1), pH depression introduced selection pressures which resulted in less diverse, acidophilic communities—nearly indistinguishable from its MH2 counterparts.

Previous work indicates that heterotrophs in MICC environments may contribute to corrosion by metabolizing microbial by-products that would otherwise inhibit neutrophilic sulfur-oxidizing populations and by producing organic acids that contribute to surface acidification [9,43]. While reduced sulfur is considered the primary energy source in this environment, organic material could serve as a heterotrophic substrate after accumulating at the concrete surface as a result of decomposing cellular debris and/or wastewater splatter. Also detected in this study were taxa related to denitrifying bacteria (*Rhodanobacter* spp.) and endosymbiotic nitrogen-fixers (*Rhizobium* spp.), indicating that the metabolic diversity of early MICC biofilms may be higher than previously hypothesized.

### Microbial succession by Sanger universal clone libraries

**Fig 4:**
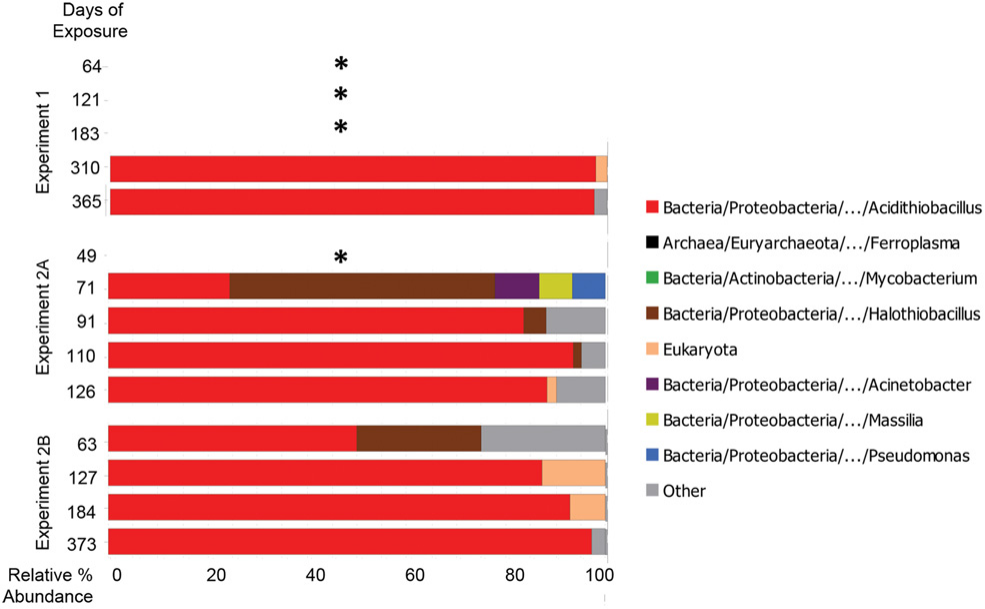
Microbial taxa observed in concrete surfaces and in corrosion products during three manhole monitoring campaigns by Sanger sequencing of universal (V4V7) rRNA clones. Taxa with greater than 1% representation in any sample are shown. Bar widths indicate relative percent abundance of microbial taxa in sample libraries; bar colors indicate taxa identity. * indicates no data due to low DNA yields.

To account for archaeal and eukaryal community members, nominally universal SSU rRNA clones also were analyzed. According to both Sanger and MiSeq methods, communities were originally dominated by *Halothiobacillus* spp., which were succeeded over time by *Acidithiobacillus* spp. (Fig. 4).

Based on Sanger sequencing, eukaryal sequences were observed in some communities exposed for 100 to 200 days in MH2 and in one community at 310 days in MH1. Sequence data were insufficient to classify eukaryal sequences into more specific groups. Previous studies have implicated acidophilic fungi in promoting the structural deterioration of corroding concrete by means of expanding hyphae [44,45].

### Congruency between culture-based model and phylogenetic results

The culture-based MICC model proposed by Islander in 1991 [5] predicted a succession from neutrophile *Halothiobacillus neapolitanus* to acidophile *Acidithiobacillus thiooxidans* over time. The bacterial succession results observed here using phylogenetic methods differs only in the presence of diverse metabolic types at early stages, including a bloom of heterotrophic types under neutral pH conditions. This similarity is rare when comparing culture-based observations to those from molecular phylogeny-based studies, because the majority of environmental microorganisms have not been successfully cultured [17]. Molecular phylogeny-based studies in other engineered systems, including drinking water distribution systems and activated sludge, have recovered markedly higher phylogenetic and metabolic diversity than was previously accounted for in culture-based studies [46]. In addition, the α-diversity observed by 16S rRNA phylogeny in this study—less than ten taxa per sample in severe corrosion—is among the lowest ever observed [38,47]. The low α-diversity may explain why microbes associated with corrosion could be previously described by culture-based observations.

## Conclusions

Corrosion of wastewater infrastructure poses an imminent problem for our society, particularly in urban settings. This study reports the longest series of longitudinal bacterial community observations reported to date in corrosion of concrete wastewater infrastructure and used high-resolution and multi-domain sequencing technology to elucidate community composition. Significant decreases in bacterial community α-diversity were observed as its composition shifted from neutrophilic to acidophilic sulfur-oxidizing microbes. This trend was concomitant with a depression of pore water pH. The findings generally support previous culture-based community succession models in concrete corrosion biofilms, with several exceptions, including a proliferation of neutrophilic heterotrophs associating with near-neutral pH conditions. Where lower diversity levels were observed, communities observed using Sanger sequencing agreed with their higher throughput counterparts recovered from an Illumina platform. Selection pressures in these environments can become sufficiently extreme to select for microbial communities which take modern concrete materials into severe corrosion states in less than a year. Given that wastewater conveyance infrastructure materials are typically designed to last upwards of 50 years, understanding the microbial progression and risk factors of this phenomenon are important to environmental sciences and the engineering field charted to protect these vital systems. This contribution to the understanding of how these communities develop can help engineers assess risk factors and help scientists elucidate community dynamics in this extreme, low-diversity ecosystem.

## Supporting Information

S1 Fig Hydrogen sulfide and carbon dioxide concentrations in manholes over time. (DOCX)

S2 Fig Surface corrosion and mass loss over time in three field experiments. (DOCX)

S1 Table Two-part analysis results. Significance determined by p-values less than 0.05.
(DOCX)
