# Supplementary material for: High-Resolution Microbial Community Succession of Microbially Induced Concrete Corrosion in Working Sanitary Manholes
Source: PLoS One. 2015 Mar 6;10(3):e0116400. doi: 10.1371/journal.pone.0116400 (PMC4352008; doi:10.1371/journal.pone.0116400)
Supplement: S1 Fig — (DOCX) [file pone.0116400.s001.docx]

**Figure S1: Hydrogen sulfide and carbon dioxide concentrations in manholes over time**
