# Supplementary material for: High-Resolution Microbial Community Succession of Microbially Induced Concrete Corrosion in Working Sanitary Manholes
Source: PLoS One. 2015 Mar 6;10(3):e0116400. doi: 10.1371/journal.pone.0116400 (PMC4352008; doi:10.1371/journal.pone.0116400)
Supplement: S1 Table — Significance determined by p-values less than 0.05. (DOCX) [file pone.0116400.s003.docx]

**Table S1: Two-part analysis results. Significance determined by p-values less than 0.05.**

| Taxa significantly more abundant  at <100 days | Taxa significantly more abundant  at >100 days |
| --- | --- |
| Gemmatimonadetes/.../Gemmatimonadaceae | Proteobacteria/.../Acidothiobacillus |
| Proteobacteria/.../Stenotrophomonas | Actinobacteria/…/Mycobacterium |
| Proteobacteria/.../Xylella |  |
| Firmicutes/.../Bacillus |  |
| Proteobacteria/.../Halothiobacillus |  |
| Nitrospirae/.../Leptospirillum |  |
| Proteobacteria/.../Ochrobactrum |  |
| Proteobacteria/.../Halothiobacillaceae |  |
| Proteobacteria/.../Arcobacter |  |
| Proteobacteria/.../Aeromonas |  |
| Proteobacteria/.../Enterobacter |  |
| Proteobacteria/.../Serratia |  |
| Proteobacteria/.../Caulobacter |  |
| Proteobacteria/.../Rhodobacteraceae |  |
| Proteobacteria/.../Massilia |  |
| Proteobacteria/.../Thiovirga |  |
| Proteobacteria/.../Cronobacter |  |
| Proteobacteria/.../Brevundimonas |  |
| Proteobacteria/.../Pseudomonas |  |
| Proteobacteria/.../Rhodanobacter |  |
| Proteobacteria/.../Klebsiella |  |
| Proteobacteria/.../Kluyvera |  |
| Proteobacteria/.../Nitrospina |  |
| Proteobacteria/.../Escherichia-Shigella |  |
